# Plastic Bronchitis: Extensive Cast Expectoration in a 6-Year-Old Boy with Fontan Circulation

**DOI:** 10.3390/diagnostics15222864

**Published:** 2025-11-12

**Authors:** Jochen Pfeifer, Martin Poryo, Peter Fries, Hashim Abdul-Khaliq

**Affiliations:** 1 Department of Pediatric Cardiology, Saarland University Medical Center, 66421 Homburg, Germany; 2 Clinic for Diagnostic and Interventional Radiology, Saarland University Medical Center, 66421 Homburg, Germany

**Keywords:** plastic bronchitis, bronchial cast, expectoration, congenital heart disease, Fontan circulation, cavopulmonary connection, bronchoscopy, complication

## Abstract

We report on a 6-year-old boy with underlying hypoplastic left heart syndrome and a total cavopulmonary connection (Fontan circulation) with a diagnosis of plastic bronchitis. After an initial good response to therapy, his productive cough became significantly stronger again. Four months later, the patient’s mother brought a preserving jar containing an extensive bronchial cast to the clinic, the size of which is rarely seen in small children. Plastic bronchitis is a rare but dreaded complication in patients with Fontan circulation as well as in infectious or inflammatory diseases; its treatment is challenging.

**Figure 1:**
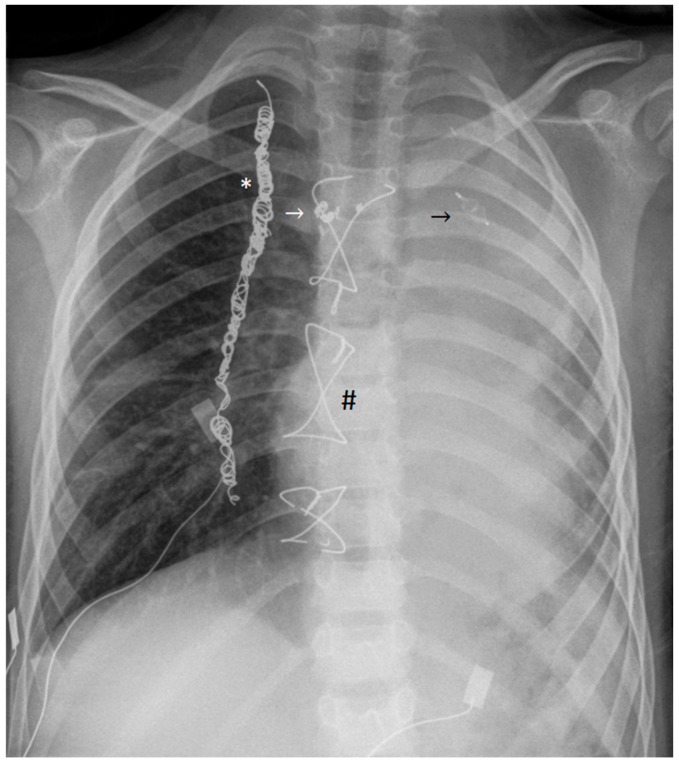
We present a case of a 6-year-old boy with hypoplastic left heart syndrome including mitral and aortic valve atresia, hypoplasia of ascending aorta, left ventricle, and atrium, as well as mild tricuspid regurgitation. At four days of age, a Norwood procedure with modified Blalock–Taussig shunt was performed. Chylothorax, wound infection, and mediastinitis complicated the postoperative course but were successfully treated. At the age of 5 months, a partial cavopulmonary connection (in the form of a bidirectional Glenn anastomosis) was established, and at the age of 4.5 years, a complete cavopulmonary connection (18 mm extracardiac Fontan conduit, 4 mm fenestration) was established. Due to the occurrence of multiple diffuse systemic-to-pulmonary collaterals originating from the right internal thoracic artery, this vessel was completely embolized with coils. Two more collaterals were selectively embolized using microcoils and a vascular plug. Other diffuse and branched arterial collaterals could not be closed due to their small size and tortuous course. He was treated with sildenafil, propranolol, lisinopril, and diuretics. The invasively measured Fontan circuit pressure was mean 10 mmHg three months earlier. First, the patient showed a good physical performance with a transcutaneous oxygen saturation of approximately 90% throughout. There were no symptoms of hypalbuminemia or protein loss, and serum albumin, immunoglobulin, and protein levels were within the normal range. Finally, the boy was admitted to the clinic with a cough that had been getting worse for several days, severe dyspnea, and reduced oxygen saturation of 55%. Chest X-ray showed extensive dystelectatic and atelectatic areas of the left lung (chest X-ray, anterior–posterior projection, showing extensive dystelectasis and atelectasis of the left lung; previously implanted wire cerclages (#), coils in the right internal thoracic artery (*), as well as microcoils and a vascular plug for embolization of systemic-to-pulmonary collaterals (arrows) are also visible). The echocardiographic examination revealed unchanged, fairly good function of the systemic ventricle with moderate tricuspid valve regurgitation and mild congestion of the inferior vena cava. The laboratory results did not reveal any significant abnormalities. Microbiological tests were negative for both viral and bacterial infections. He underwent bronchoscopy with lavage and removal of several fractions of rubber-like fibrous casts from the left bronchi, which histologically contained mucin, fibrin, lymphocytes, and macrophages, leading to the diagnosis of plastic bronchitis. Subsequent treatment in the pediatric intensive care unit included mechanical ventilation for 10 days, and inhaled mucolytic, fibrinolytic, and bronchodilator therapy, namely dornase alpha, hypertonic saline, recombinant tissue-type plasminogen activator, salbutamol, and ipratropium bromide. In addition, positioning therapy and repeated suctioning of the airways were performed. The boy’s condition improved significantly. He was discharged after three weeks and continued with inhaled mucolytic and steroid therapy (saline, aerosolized heparin, budesonide, and dornase intermittently), anticongestive medication, and physiotherapy at home. In addition, treatment for pulmonary arterial hypertension (sildenafil) was continued.

**Figure 2:**
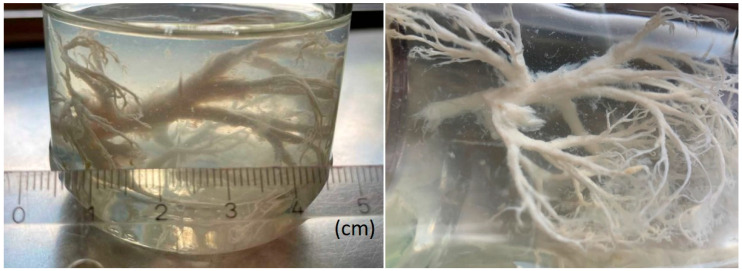
Four months later, the boy’s mother presented a preserving jar containing a highly ramified, whitish cast that the boy had expectorated due to persistent plastic bronchitis (photographs (two views) of the jar containing the cast in aqueous solution). The expectoration represented a coherent three-dimensional cast of the bronchial system of almost an entire lung side with a maximum length of approximately 8 cm. Also referred to as cast bronchitis, plastic bronchitis is a rare but severe complication of various diseases with an incidence of about 4% in Fontan patients [1]. Its symptoms are chronic cough, hypoxemia, and expectoration of proteinaceous casts, which mainly consist of fibrin and leucocytes. Cases involving expectoration of extensive bronchial casts have previously been described [2,3,4,5,6,7,8], but they are very rare to this extent in young children. In complex congenital heart diseases, failing Fontan circulation and systemic-to-pulmonary collaterals typically promote the disease. Further risk factors include chylothorax, ascites, and prolonged chest drainage after surgery [9]. Male patients are more frequently affected [10]. The exact etiology of plastic bronchitis is still unknown. The most probable pathophysiological causes are increased systemic venous pressure, lymphostasis, and retrograde intrathoracic lymph flow; increased capillary permeability due to chronic inflammation promotes the leakage of protein-containing fluid into the bronchi and the formation of casts. The loss of intravascular proteins and hypoalbuminemia lead to a vicious circle with a further reduction in oncotic pressure and resulting lymphatic leakage [9,11,12]. Plastic bronchitis in Fontan patients has serious prognostic implications, as the 5-year mortality rate is reportedly up to 50% [13]. Treatment is challenging and not standardized. A combination of anti-obstructive and anti-inflammatory treatment, as well as optimization of the Fontan physiology, appears to be the most promising strategy [14]. In addition to the above-mentioned fibrinolytic and mucolytic medication, which were used in our case, N-acetylcysteine, urokinase, or alpha-chymotrypsin as well as pulmonary vasodilators, octreotide, and low-fat diet are therapeutic options. Surgical and interventional procedures may include the creation of a Fontan fenestration (if missing) or Fontan takedown as well as embolization of systemic-to-pulmonary collateral arteries [14,15,16,17,18]. Selective percutaneous lymphatic embolization, for example, using n-butyl-2-cyanoacrylate, vascular plugs, or microcoils [9,19,20,21], or surgical ligation of the thoracic duct [22], may be considered in the absence of a therapeutic response. Although precise data are lacking, the risk of refractory or recurrent plastic bronchitis appears to be high. Compared to previously reported pediatric cases, ours is a typical case of plastic bronchitis, as it involves a male Fontan patient who expectorated characteristic casts. Nevertheless, the huge casts described, which the patient’s mother presented in a jar, are rarely seen in young children. The altered lymphatic drainage following chylothorax after the first operation, the multiple collaterals, as well as the tricuspid insufficiency may have contributed to the development of plastic bronchitis in our patient. Rigorous embolization of arterial systemic-to-pulmonary collaterals and percutaneous lymph embolization must be considered. However, recurrence despite extensive therapy can occur frequently. Other possible diseases associated with plastic bronchitis are asthma, *Mycoplasma pneumoniae* pneumonia, chronic inflammatory or infectious diseases, previous surgical procedures, and other chronic or severe systemic diseases [15,23,24]. The case presented shows that plastic bronchitis is a very difficult complication to treat in patients with Fontan physiology. Even in young children and during treatment, severe cast expectoration may occur. Combined therapy with inhalation, respiratory therapy, and the optimization of Fontan circulation is the most effective approach.

## Data Availability

Data are contained within the article.
